# Comparison of CRISPR–Cas Immune Systems in Healthcare-Related Pathogens

**DOI:** 10.3389/fmicb.2021.758782

**Published:** 2021-10-25

**Authors:** Kate Mortensen, Tony J. Lam, Yuzhen Ye

**Affiliations:** Luddy School of Informatics, Computing and Engineering, Indiana University, Bloomington, IN, United States

**Keywords:** CRISPR–Cas, spacer, protospacer, AMR genes, ESKAPE pathogens

## Abstract

The ESKAPE pathogens (*Enterococcus faecium, Staphylococcus aureus, Klebsiella pneumoniae, Acinetobacter baumannii, Pseudomonas aeruginosa*, and *Enterobacter* species) and *Clostridium difficile* have been identified as the leading global cause of multidrug-resistant bacterial infections in hospitals. CRISPR–Cas systems are bacterial immune systems, empowering the bacteria with defense against invasive mobile genetic elements that may carry the antimicrobial resistance (AMR) genes, among others. On the other hand, the CRISPR–Cas systems are themselves mobile. In this study, we annotated and compared the CRISPR–Cas systems in these pathogens, utilizing their publicly available large numbers of sequenced genomes (e.g., there are more than 12 thousands of *S. aureus* genomes). The presence of CRISPR–Cas systems showed a very broad spectrum in these pathogens: *S. aureus* has the least tendency of obtaining the CRISPR–Cas systems with only 0.55% of its isolates containing CRISPR–Cas systems, whereas isolates of *C. difficile* we analyzed have CRISPR–Cas systems each having multiple CRISPRs. Statistical tests show that CRISPR–Cas containing isolates tend to have more AMRs for four of the pathogens (*A. baumannii, E. faecium, P. aeruginosa*, and *S. aureus*). We made available all the annotated CRISPR–Cas systems in these pathogens with visualization at a website (https://omics.informatics.indiana.edu/CRISPRone/pathogen), which we believe will be an important resource for studying the pathogens and their arms-race with invaders mediated through the CRISPR–Cas systems, and for developing potential clinical applications of the CRISPR–Cas systems for battles against the antibiotic resistant pathogens.

## 1. Introduction

ESKAPE pathogens are the primary cause of nosocomial infections (infections contracted from a healthcare setting) and are of global concern due to the increasing emergence of multi-drug resistant (MDR) bacteria (Zohra et al., 2021). The term ESKAPE pathogens was first coined by Rice (Rice, 2008) and originally included *Staphylococcus aureus, Enterococcus faecium, Klebsiella pneumoniae, Acinetobacter baumanii, Pseudomonas aeruginosa*, and *Enterobacter* spp. Recently, *Clostridium difficile* is just one of the suggested additions to this collection with the increase of antimicrobial-resistant pathogens in the past decade (Peterson, 2009). The acronym, ESKAPE, conveniently emphasizes the severity of these virulent species and describes their method of antimicrobial evasion. They are now attributed to the majority of US hospital infections and effectively “escape” the limited bank of available antibiotics by acquiring antimicrobial resistance (AMR) genes. The threat of multi-drug resistant bugs is constant and has fueled a variety of research and surveillance efforts (Zohra et al., 2021).

The slow state of antibiotic development, antimicrobial therapies, and lack of coordinated global surveillance has rendered the ESKAPE pathogens particularly nefarious (De Oliveira et al., 2020). A myriad of alternative therapies has been presented (e.g., nanotechnology, antimicrobial peptides, and phage therapy), many have attractive advantages over antibiotics but they too are not without limitations (Sharma et al., 2016; Munir et al., 2020). These therapeutic methods range in functional diversity and have the potential to compliment both new and traditional treatments. Phage therapy is especially intriguing in that phage action a more targeted attack against pathogens while maintaining organic compatibility with the host, unlike the reputation of antibiotics (Mulani et al., 2019). While phage therapy is promising, the mischievous nature of bacterial defense mechanisms can curb success. Fundamental research around pathogen defense mechanisms and immunity, such as CRISPR–Cas systems, are key in understanding how to disarm pathogens, successful therapies, and prevent horizontal gene transfer of AMR genes.

CRISPR–Cas systems are RNA-mediated defense systems that act against invasive DNA/RNA sequences commonly found in Bacteria and Archaea species. While these complex systems can inhibit phage attacks and invasion of other foreign nucleic matter, they remain adaptive through their ability to acquire immunological memory of past encounters to foreign mobile genetic elements. On the other hand, invaders have various mechanisms, including anti-CRISPR (Pawluk et al., 2018) to circumvent CRISPR–Cas, and this dynamic interplay between defense and adaptive survival may allow for horizontal gene transfer of AMR genes (Malone et al., 2021). A typical CRISPR–Cas system is usually composed of genomic regions called clustered regularly inter-spaced short palindromic repeats (CRISPRs), who's array is comprised of spacer sequences flanked by repeat sequences, and a set of CRISPR-associated (Cas) proteins. Generally, spacer sequences, acquired from foreign nucleic acids, are separated by 24 to 47-bp repeats (Zhang and Ye, 2017). Cas proteins, encoded by *cas* genes, play important roles in all stages of the defense process including adaptation, expression, and interference. Based on the specific combination of *cas* genes, CRISPR–Cas immune systems can be divided into two classes (I and II), each containing several types and subtypes (Makarova et al., 2020). These systems have recently been engineered to achieve guided genome editing and gene expression regulation in many different organisms, including mammalian cells (Hendriks et al., 2020). Such genetic engineering advances have driven innovative applications in several fields, from basic biology to biotechnology and medicine.

There are a variety of existing studies around CRISPR–Cas systems and ESKAPE genomes, however, these data are not available for download and visualization. CRISPR–Cas systems of one major type (type I) in three sub-types (F, E, and C) have been identified in *P. aeruginosa* (van Belkum et al., 2015), and they were shown to restrict horizontal gene transfer in this species (Wheatley and MacLean, 2021). Studies have also previously explored the CRIPSR-Cas dynamics in *C. difficile* (Maikova et al., 2018). Recent advances in sequencing technology have boosted the applications of whole genome sequencing (WGS) in clinical settings. Examples include pathogen transmission tracking (Grad and Lipsitch, 2014; Bentley and Parkhill, 2015), rapid identification of virulence factors in outbreak analysis (Gardy et al., 2011; Cáceres et al., 2013; Gilchrist et al., 2015), and the characterization of evolutionary dynamics of pathogens in healthcare systems (Hsu et al., 2015). *S. aureus* isolates have certainly been a focus for molecular epidemiology well within the past decade and beyond.

As ESKAPE pathogens and MDR bacteria become increasingly prevalent, research into the evolutionary and environmental dynamics of these pathogenic species becomes increasingly important. In this paper, we systematically analyze the large number of publicly available ESKAPE genomes, focusing on the CRISPR–Cas immune systems and AMR genes and their relationship, and present our findings as an important resource to study ESKAPE pathogens, their potential invaders, and to develop potential clinical alternatives to against antibiotic resistant pathogens.

## 2. Materials and Methods

### 2.1. Genome Sequences and Pangenome Analysis

We analyzed the CRISPR–Cas systems for all members of ESKAPE pathogens except for *Enterobacter* spp. due to the large number of species which fall into this genus. Additionally, we've included *C. difficile* into our analysis due to its increasing role in nosocomial infections and ability to evade antibiotic treatment. Hereinafter, we will collectively refer to the members of these pathogens as ESKAPE+C for simplicity.

We downloaded all the complete and draft genomes for *E. faecium, S. aureus, K. pneumoniae, A. baumannii, P. aeruginosa*, and *C. difficile* from the NCBI RefSeq FTP Database (as of Jan 18, 2021). Table 1 summarizes the number of genomes for each pathogen and their lengths. Lists of the genomes we analyzed can be found at the CRISPR-pathogen website at https://omics.informatics.indiana.edu/CRISPRone/pathogen.

**Table 1:** Table summary of the number of ESKAPE+C genomes analyzed.

**Species**	**#-genomes**	**#-complete**	**#-draft**	**Median genome**
				**size (Mb)**
*A baumannii*	4,893	246	4,647	3.97
*C. difficile*	1,932	66	1,872	4.16
*E. faecium*	2,223	191	2,032	2.92
*K. pneumoniae*	10,053	755	9,298	5.60
*P. aeruginosa*	5,576	289	5,287	6.60
*S. aureus*	12,212	599	11,613	2.83

### 2.2. Characterization of CRISPR–Cas Systems

**Figure 1:**
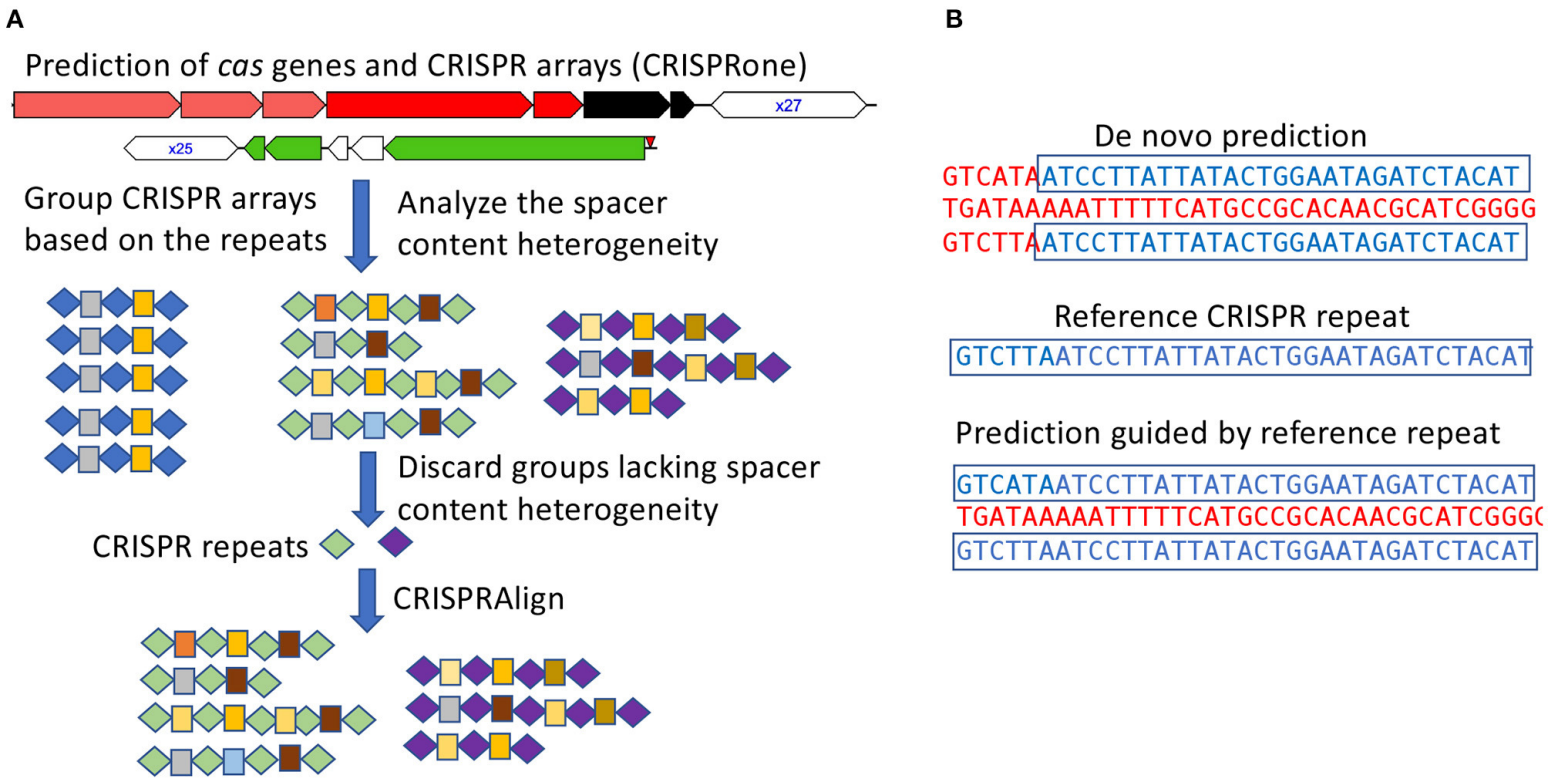
Annotation of CRISPR–Cas systems in all genomes of the pathogens. **(A)** CRISPR arrays identified from all isolates for a pathogen are analyzed in groups such that false arrays can be identified and discarded; In this illustration, repeats and spacers are represented as diamonds and boxes, respectively, and the same repeats/spacers are highlighted in the same color. **(B)** using checked CRISPR repeats (as the references) to guide the characterization of CRISPR arrays.

CRISPR–Cas systems were first identified using CRISPRone (Zhang and Ye, 2017). Briefly, CRISPRone predicts *cas* genes by searching putative proteins against Hidden Markov Models (HMM) for the different Cas proteins and uses metaCRT for *de novo* identification of CRISPR arrays (Figure 1A). The annotated compositions of the *cas* genes are then used to infer the CRISPR–Cas system types and sub-types.

To facilitate the analysis of numerous CRISPR arrays identified from isolates of the same species and achieve a more refined characterization of the observed CRISPR arrays, the CRISPR arrays were grouped according to their repeat sequences such that arrays containing similar repeats were grouped together. Taking CRISPR array grouped by repeat sequences, the spacers from a group of arrays were clustered to identify identical or near identical spacer sequences shared across different arrays. Spacer sequences were clustered with CD-HIT-EST (Li and Godzik, 2006) at 85% sequence identity, allowing for spacer clusters to contain slight sequence variation due to sequencing error or mutation.

In some instances, it may be difficult to filter out false positive arrays and inactive CRISPR–Cas systems. To address this issue—which becomes serious when analyzing thousands of isolates for a species—we propose a metric to measure the heterogeneity of spacer contents among CRISPRs to proximate turnover of spacers and identify active CRISPR–Cas systems. We define the spacer content heterogeneity score as:


(1) $$\text{Spacer Heterogeneity}=\frac{m-max(c_{i})}{\sum_{i}^{n}c_{i}-max(c_{i})}$$

Where *n* is defined as the number CRISPR arrays, with each CRISPR array containing *c*_1_, *c*_2_,…, *c*_*n*_ unique spacers (in some rare cases, CRISPR arrays may contain multiple copies of the same spacer, which will be considered as one spacer). And *m* denotes the number of unique spacers found from all *n* arrays combined.

The heterogeneity score ranges from 0 to 1, with 0 indicating no spacer heterogeneity and 1 for the highest spacer content heterogeneity among the CRISPR arrays. For example, assume there are two arrays each containing 10 spacers. If the two arrays share no spacers, the heterogeneity score is 1; if the two arrays share the same spacer contents, the heterogeneity score is 0; and if the two arrays share half of their spacers (e.g., five spacers from array 1 is the same as five spacers from array 2), then the heterogeneity score is 0.5 $\left(\frac{15-10}{20-10}\right)$. CRISPR groups that lack spacer content heterogeneity and have no adjacent *cas* genes are considered inactive or false positive, and discarded from further analysis.

Additionally, *de novo* prediction of CRISPR arrays can sometimes be challenging due to mutations in repeats. These mutations may prevent *de novo* prediction software from properly accounting for boundaries of the CRISPR array or cause the CRISPR array to be missed in instances of short CRISPR arrays (Figure 1B). To account for potential erroneous or missed predictions of CRISPR arrays, a post-processing step was preformed to refine repeat-spacer boundaries. CRISPR array repeats were extracted from groups of valid CRISPR arrays (with spacer content heterogeneity as described above), clustered, and finally manually checked to establish a set of high confident reference repeats. These reference repeats were then used as input for CRISPRAlign (Rho et al., 2012), to identify CRISPR arrays in the genomes that contain these repeats (flanking spacers).

### 2.3. Characterization of the AMR Genes

To identify antibiotic resistance genes, genomes were analyzed using NCBI's AMRFinderPlus (Feldgarden et al., 2021) where coding sequences (CDS) were predicted, and antimicrobial resistance (AMR) genes were annotated through a combination of HMMER (Eddy, 1998) and BLASTP (Altschul et al., 1990) searches. AMRFinderPlus searches against a curated database of AMR genes and protein profile HMMs. Additionally, a hierarchical tree of AMR protein families and a custom ruleset is used by AMRFinderPlus to filter results, and generate names and coordinates for identified AMR genes.

### 2.4. Mobile Genetic Element Databases

A collection of mobile genetic element (MGE) databases (i.e., phage and plasmid databases) were collected for Host-MGE interaction analysis. Phage databases include the Gut Phage Database (Camarillo-Guerrero et al., 2021) (GPD), MicrobeVersusPhage (Gao et al., 2018) (MVP) database, and the reference viral database (Goodacre et al., 2018) (RVDB). Plasmid databases referenced the Comprehensive and Complete Plasmid Database (Douarre et al., 2020) (COMPASS), and PLSDB (Galata et al., 2019). Collectively, these databases encompass phage and plasmids sequences mined from the NCBI reference database, NCBI nucleotide database, metagenome assemblies, and prophages identified in prokaryotic genomes.

### 2.5. Identification of CRISPR Targets

Spacer sequences were first extracted from predicted CRISPR arrays. Putative MGE associations were linked to specific spacer sequences by querying spacer sequences against the collection of MGE databases via BLASTN (Altschul et al., 1990). BLAST hits that had greater than 90% sequence identity, query coverage per hsp greater than 80%, and an *e*-value of less than 0.001 were retained for downstream processing. To remove possible duplicate hits due to overlapping genomes in different databases, subject genomes identified through blast were dereplicated using dRep (Olm et al., 2017) with default parameters, and duplicated genomes were filtered from blast results. Remaining results were considered to be positive associations between a given CRISPR spacer and their putative targets with matching protospacers.

A CRISPR-based MGE-host network was then constructed using spacer and MGE associations. A spacer to MGE edge was constructed in a network if a given spacer has a matching protospacer found in a phage or plasmid in the MGE databases. All visualizations and manual inspections of constructed networks were performed using Cytoscape (Shannon et al., 2003).

## 3. Results

### 3.1. CRISPR–Cas Systems of ESKAPE+C Pathogens

**Figure 2:**
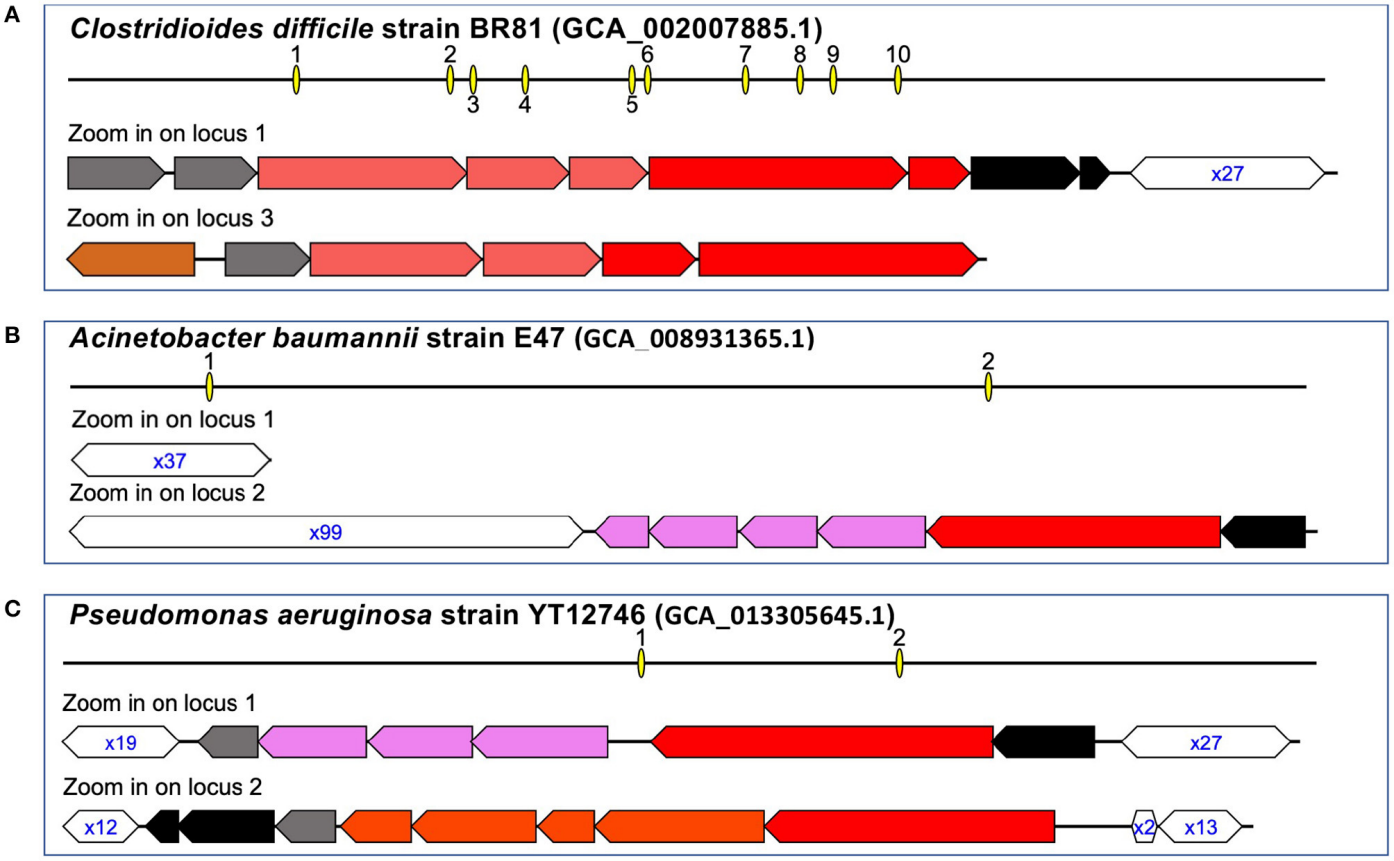
Representative CRISPR–Cas systems in the pathogens. The arrows represent genes (the annotations of the genes can be found in our website), and the open hexagons represent the CRISPR arrays, with the text inside the hexagons showing the copy number of the repeat found in the corresponding CRISPR, e.g., x27 in **(A)** indicates the corresponding array contains 27 copies of the repeat (spanning 26 spacers). Interactive visualizations of the representative CRISPR–Cas systems with detailed information of the *cas* genes and the organization of the repeat-spacer units in the CRISPRs are available at the supplementary website. Here we show CRISPR-Cas systems in three genomes: *C. difficile* strain BR81 **(A)**, *A. baumannii* train E47 **(B)**, and *P. aeruginosa* strain YT12746 **(C)**.

Table 2 summarizes the types and prevalence of CRISPR–Cas systems found in ESKAPE+C genomes. The ESKAPE+C species that were analyzed showed varying degrees of CRISPR–Cas system prevalence, as well as varying types of CRISPR–Cas systems. All *C. difficile* genomes analyzed contained a Type I-B CRISPR–Cas system. Additionally, *C. difficile* genomes were also found to have excessive number of CRISPR arrays, sometimes these arrays were accompanied by *cas* genes but oftentimes not. For example, *C. difficile strain BR81* (Figure 2A) was found to have 10 CRISPR loci, but one out of 10 CRISPR loci contains both *cas* genes and a CRISPR array; one only contains *cas* genes, and the rest only contain CRISPR arrays. We note a *C. difficile* isolate (GCA_000211235.1_ASM21123v1) contains a phage with a Type-I CRISPR–Cas system (but no CRISPR array), shedding light on the mobile nature of the CRISPR–Cas systems. In comparison, only about 15.76% of *A. baumannii* genomes were found to have Type I-F CRISPR–Cas systems (Figure 2B). In *P. aeruginosa* only Type-I CRISPR–Cas systems were found in this species with a prevalence of 60.33% (Figure 2C). Of the Type-I CRISPR–Cas systems, there contained three sub-types: Type I-C, Type I-E, and Type I-F. In about 91.56% of *E. faecium* genomes contained CRISPR–Cas systems, however about 54.54% of genomes of those CRISPRs are considered orphan CRISPRs meaning the CRISPR–Cas systems are inactive due to the lack of associating Cas proteins. *E. faecium* was found to contain Type II-A CRISPR–Cas systems with a low prevalence rate of 2.47% among all reference genomes.

**Table 2:** Main types of CRISPR–Cas systems found in the pathogens and their characteristics.

**Species**	**CRISPR–Cas**	**Prevalence (%)**	**Notes**
*A. baumannii*	I-F	15.76	One single CRISPR array or multiple ones
*C. difficile*	I-B	100.00	Excessive CRISPR arrays
*E. faecium*	II-A	2.47	Type-II system in a small number of isolates
*K. pneumoniae*	I-E, unk-plasmid	32.39	Plasmids have CRISPR–Cas systems
*P. aeruginosa*	I (-C, -E, -F)	60.33	One of the three subtypes (-C, -E, or -F) in each isolate
*S. aureus*	II (-C), III (-A)	0.55	Both types are rare, II found in only one isolate

In *K. pneumoniae*, 32.39% of genomes were found to contain Type I-E CRISPR–Cas systems. Additionally, *K. pneumoniae* was also found to contain plasmids that have a CRISPR–Cas system: CRISPR–Cas systems were found in the plasmids of a total of 25 isolates of this species (e.g., *K. pneumoniae* strain KPNIH48, GCA_002935085.1_ASM293508v1). Out of all ESKAPE+C genomes, *S. aureus* contained the least CRISPR–Cas systems with a prevalence of 0.55% of genomes. While both Type II-C and Type III-A CRISPR–Cas systems were observed to be present in *S. aureus* genomes, both systems were quite rare, in particular, Type II-C CRISPR–Cas systems which was only observed in a singular genome.

**Figure 3:**
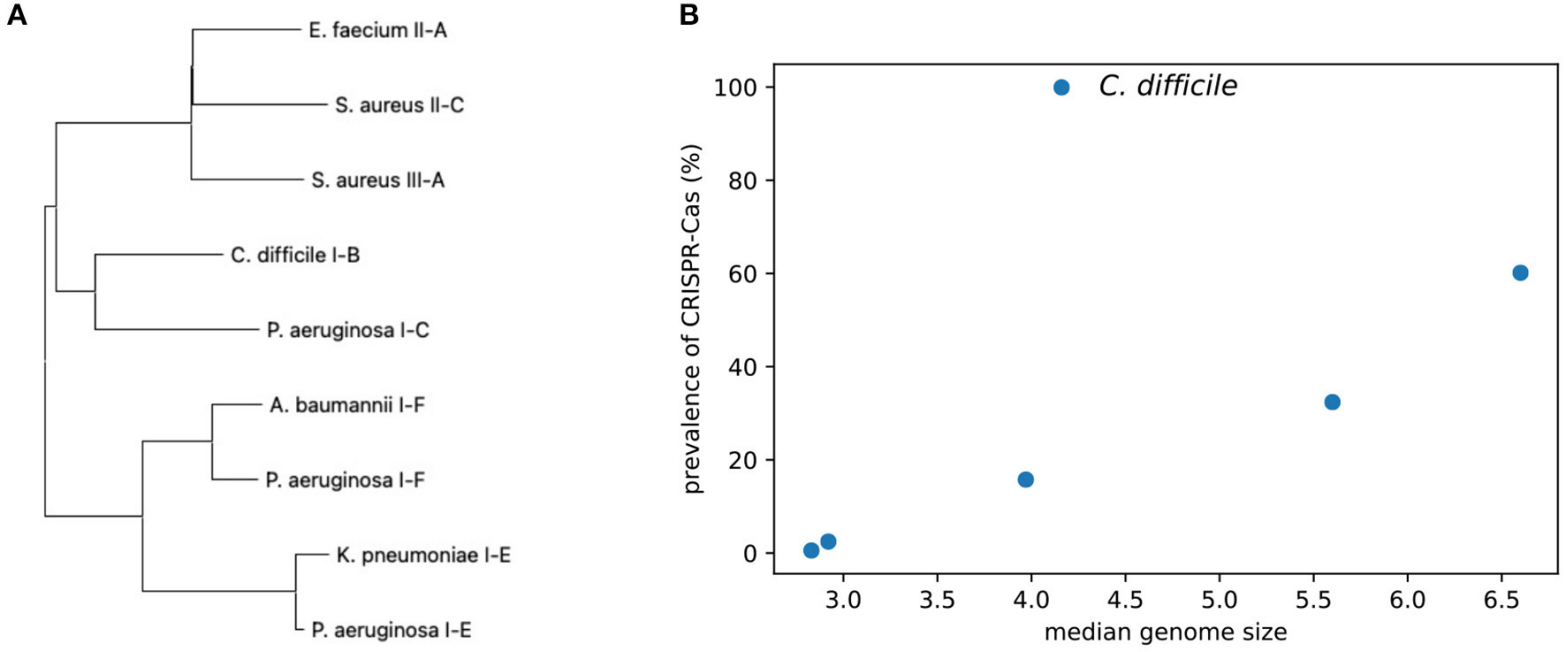
Comparison of the CRISPR–Cas systems identified from the ESKAPE+C pathogens. **(A)** A phylogenetic tree of the universal *cas1* genes associated with CRISPR–Cas systems. **(B)** Graph comparing the prevalence of CRISPR–Cas systems among ESKAPE+C pathogens and their genome size (Mb).

As shown in Figure 2 and Table 2, the pathogens harbor different types of CRISPR–Cas systems and some contain multiple (sub)types. Since different types of CRISPR–Cas systems involve different *cas* genes, we used the universal *cas1* genes to study the relationship of the different types of CRISPR–Cas systems. We used MEGA (Hall, 2013) to create a maximum likelihood tree of the *cas1* genes using the MUSCLE (Edgar, 2004) multiple alignment of the protein sequences of the *cas1* genes. Not surprisingly, the tree (Figure 3A) shows that *cas* genes involved in the same (sub)type of CRISPR–Cas systems from different species tend to group together. For example, the *cas1* genes of the three subtypes of CRISPR–Cas systems in *P. aeruginosa* are in three clades, with type I-E *cas1* gene grouping with *cas1* gene of the same type in *K. pneumoniae*.

The pathogens have a broad spectrum of CRISPR–Cas prevalence. As shown in Figure 3B, there is a general trend that isolates of the pathogens with larger genomes (e.g., *P. aeruginosa*) tend to have CRISPR–Cas systems as compared to pathogens with smaller genomes (e.g., *S. aureus*). However, the correlation is only marginally significant (Spearman correlation = 0.83, *p* = 0.042), probably due to *C. difficile* who's isolates we analyzed all contained CRISPR–Cas systems.

### 3.2. Distribution of AMR Genes in Pathogens and Their Correlation With CRISPR–Cas Systems

**Figure 4:**
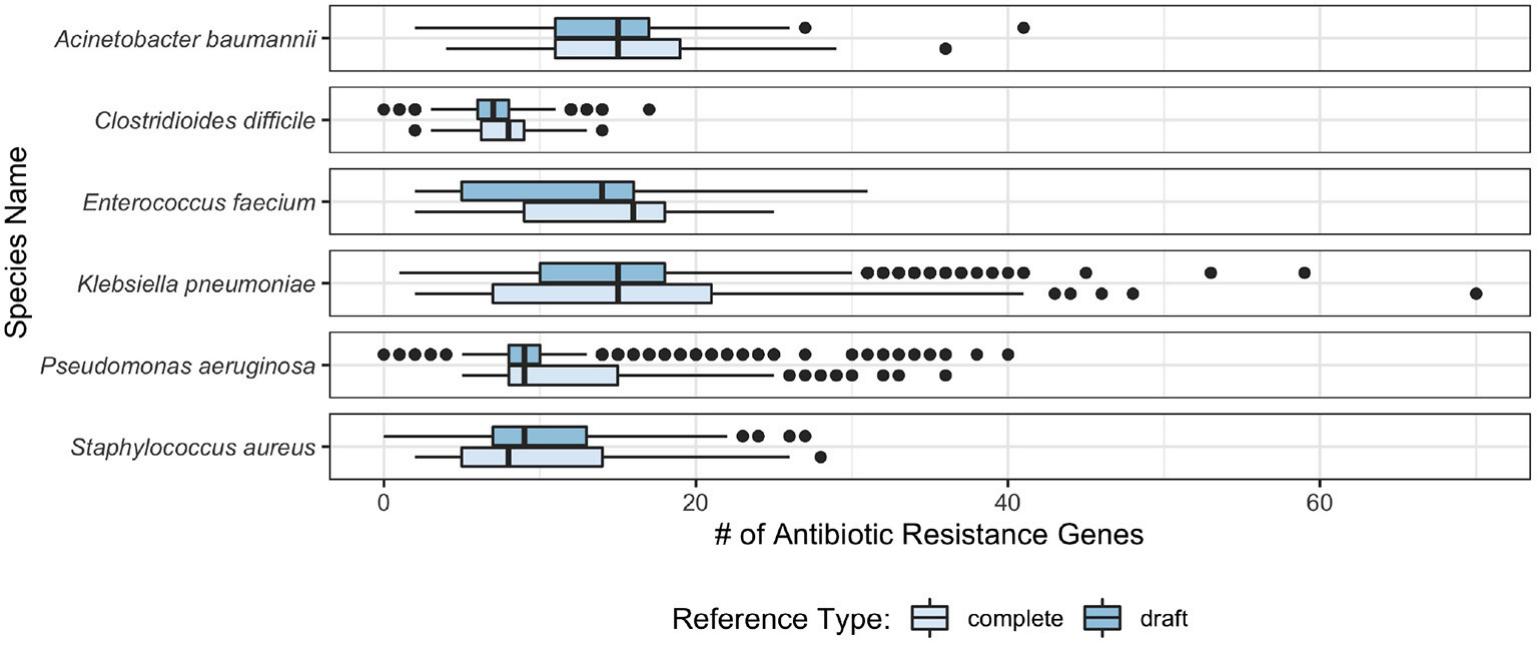
Box plot of the number of AMR genes found per genome in ESKAPE+C pathogens in complete and draft genomes.

**Figure 5:**
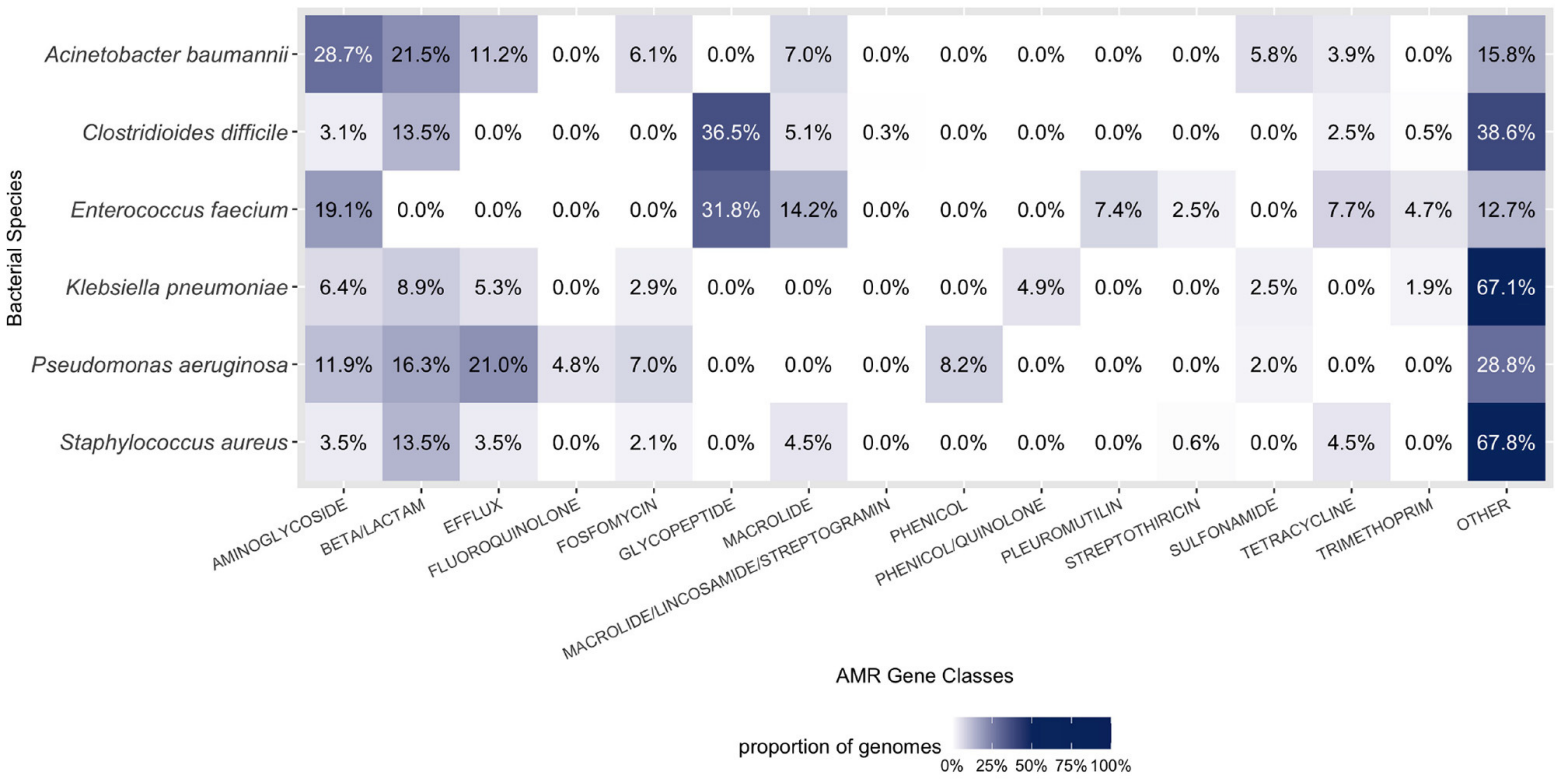
Composition of AMR gene classes by species. A large proportion of each ESKAPE+C pathogen's genome consists of AMR genes. Only the top six abundant AMR gene classes for each species were considered in the figure above. Percentages were calculated in context of all gene classes, both AMR and otherwise.

Observing the distribution of AMR genes for ESKAPE+C genomes (Figure 4), we see that *A. baumannii* and *K. pneumoniae* genomes contain on average the largest number of AMR genes, 13.9 and 14.7 per genome, respectively. Unsurprisingly, no significant variations of AMR gene content were observed between complete and draft reference genomes. As shown in Figure 5, different pathogens tend to have different types of AMR genes. Similarities and differences in the distribution of antibiotic resistance genes are notable between pathogens. While some AMR gene classes are shared by all (e.g., aminoglycoside), other classes may only be present in a subset of these species (e.g., phenicol in *P. aeruginosa* only, pleuromutilin in *E. faecium* only, and glycopeptide class found in *E. faecium* and *C. difficile*). One notable difference is the distribution of the glycopeptide AMR gene class across species, constituting 36.5 and 31.8% of all AMR genes in the pangenomes of *C. difficile* and *E. faecium*, respectively, but are absent in the remaining ESKAPE+C pathogens.

Statistical tests (Mann–Whitney *U*-test and Kruskal Wallis test) between the presence of CRISPR–Cas systems and the abundance of AMR genes were shown to be statistically significant for four pathogens, *A. baumannii, P. aeruginosa, S. aureus*, and *E. faecium*, where the CRISPR–Cas containing isolates tend to have fewer AMR genes than their CRISPR–Cas absent counterparts (Table 3). Non-parametric tests were used due to non-normal distribution of AMR genes between isolates. We note that *K. pneumoniae* was found to have a (non-significant) greater number of AMR genes in CRISPR containing isolates compared to isolates without CRISPR–Cas system. This observation can partly be explained by the presence of CRISPR–Cas carrying plasmids, which also carry AMR genes, increasing the correlation between AMR genes and CRISPR–Cas systems seen in *K. pneumonia*.

**Table 3:** Correlation (or lack of correlation) between AMR genes and CRISPR–Cas systems.

**Species**	**AMR (CRISPR+)**	**AMR (CRISPR-)**	**Mann–Whitney U**	**Kruskal Wallis**	**Correlation**
*A baumannii*	9.8/11	12.1/14	1.23e-27 (less)	2.46e-27	Yes
*E. faecium*	3.8/3	10.5/13	8.77e-13 (less)	1.73e-12	Yes
*K. pneumoniae*	13.4/14	12.9/14	0.056 (greater)	0.113	No
*P. aeruginosa*	9.3/9	10.2/9	4.7e-48 (less)	9.5e-48	Yes
*S. aureus*	4.3/0	6.1/6	0.0079 (less)	0.0157	Yes

*AMR (CRISPR+) and AMR (CRISPR-) list the mean/median number of AMR genes in the isolates with and without CRISPR–Cas systems, respectively. The Mann–Whitney U and the Kruskal Wallis columns list the p-values of the corresponding tests. For Mann–Whitney U-test, “less” and “greater” indicate the test was applied to test if the CRISPR–Cas containing isolates have fewer, or more AMR genes as compared to CRISPR–Cas lacking isolates, respectively. We didn't include C. difficile in this table since all its isolates contain CRISPR–Cas systems*.

*E. faecium* has the largest difference of AMR genes among their isolates depending on if the isolates have CRISPR–Cas systems or not. The median number of AMR genes predicted in *E. faecium* isolates that lack CRISPR–Cas system is 13, whereas the CRISPR–Cas containing isolates have far more fewer AMR genes (the median is 3), suggesting the CRISPR–Cas systems could be an effective barrier to the antibiotic resistance dissemination related to *E. faecium*.

### 3.3. Identification of Invaders Based on CRISPR Spacers: *K. pneumonia* as a Case Study

**Figure 6:**
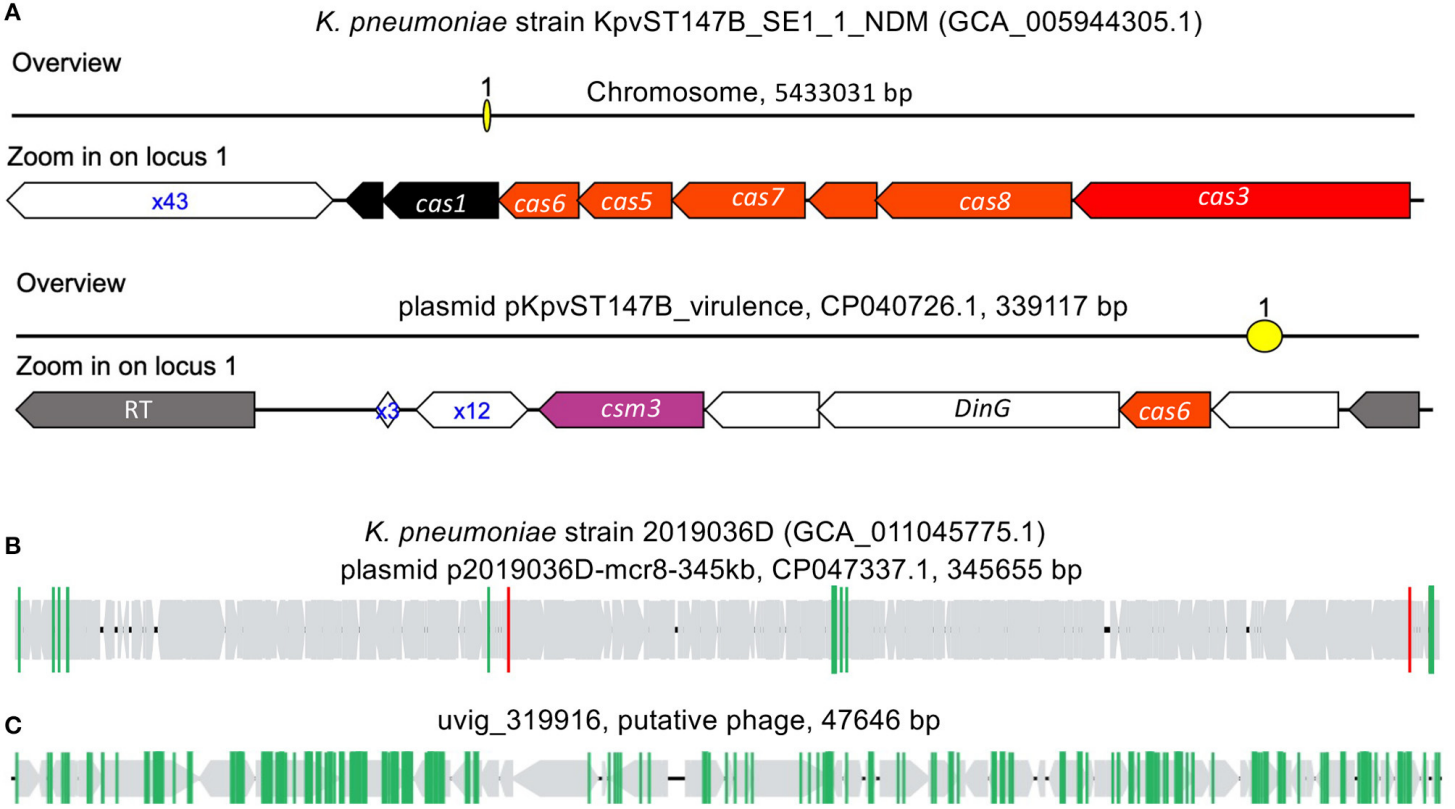
CRISPR–Cas systems in *K. pneumoniae* genomes. **(A)** type I-E CRISPR–Cas system found in the chromosome of *K. pneumoniae* strain KpvST147B_SE1_1_NDM, and the other CRISPR–Cas (likely type IV) in the plasmid. **(B,C)** are selected examples of plasmid **(B)** and phage **(C)** invaders that contain protospacers matching spacers found in *K. pneumoniae*'s CRISPR–Cas systems. In **(B,C)**, the gray arrows represent the genes predicted in the invaders, and the vertical lines each represent a protospacer.

To better understand the dynamics between MGE and their hosts, as CRISPR–Cas systems are adaptive immune systems with mechanisms for acquired immunological memory, CRISPR array spacers can be used to identify putative interactions with past encounters with MGEs. Here we analyzed the CRISPR–Cas systems of *K. pneumoniae* to demonstrate the use of CRISPR–Cas systems and better understand its interactions with MGEs (e.g., phages and plasmids). We focus our analysis on *K. pneumonia* because it has sizeable CRISPR–Cas prevalence (32.39%) and it is second in rank of ESKAPE+C species with the largest number of available reference genomes (10,053 samples); in contrast to *S. aureus* which has the largest number of available reference genomes (12,212) but only has a CRISPR–Cas prevalence of 0.55%. In *K. pneumoniae*, ~32.39% of genomes were found to have Type I-E CRISPR–Cas systems; a representative Type I-E CRISPR–Cas system typically found in *K. pneumoniae* is shown on Figure 6A. CRISPR–Cas carrying plasmids have also been found to be associated with *K. pneumoniae* genomes (Figure 6A), which CRISPRone has annotated as a Type IV CRISPR–Cas system; CRISPR carrying, antibiotic resistant plasmids have previously been reported by Kamruzzaman et al. (Kamruzzaman and Iredell, 2020). It should be noted that while the CRISPR–Cas systems found in plasmids are annotated as a Type IV CRISPR–Cas system, only a single gene specific to Type IV systems, *divG*, is found within the CRISPR–Cas locus.

By matching *K. pneumoniae* spacers to an MGE database, we were able to identify putative CRISPR targets of invader MGEs. *K. pneumoniae* CRISPRs were found to favor targeting phage over plasmids, with 3,384 spacer sequences having matched to protospacers found in 4,755 phages and 1,765 plasmids. In many instances, protospacer containing phages and plasmids contained more than one protospacer. An example of putative plasmid invader (Figure 6B), plasmid pKpvST147B_virulence (CP047337.1, found in *K. pneumoniae* strain 2019036D, which doesn't contain any CRISPR–Cas systems in its chromosome and associated plasmids) was found to contain multiple protospacers. Among the protospacers, two (highlighted in red vertical lines in Figure 6B) match the spacers in the CRISPR array carried by the plasmid of *K. pneumoniae* strain KpvST147B_SE1_1_NDM (Figure 6A). In phages, the number of protospacers found per protospacer containing genome was usually greater than that of plasmids. An example of a putative phage invader (Figure 6C), uvig_319916, was found to have 158 protospacers; all of which matched to spacers found in CRISPR arrays in *K. pneumoniae* Type I-E CRISPR–Cas systems. The putative phage genome, uvig_319916, is a putative phage part of the GutPhageDatabase (Camarillo-Guerrero et al., 2021) and was recovered from the gut metagenome assembly of SRR413656.

### 3.4. Interaction Network of *K. pneumoniae* With Its Invaders

**Figure 7:**
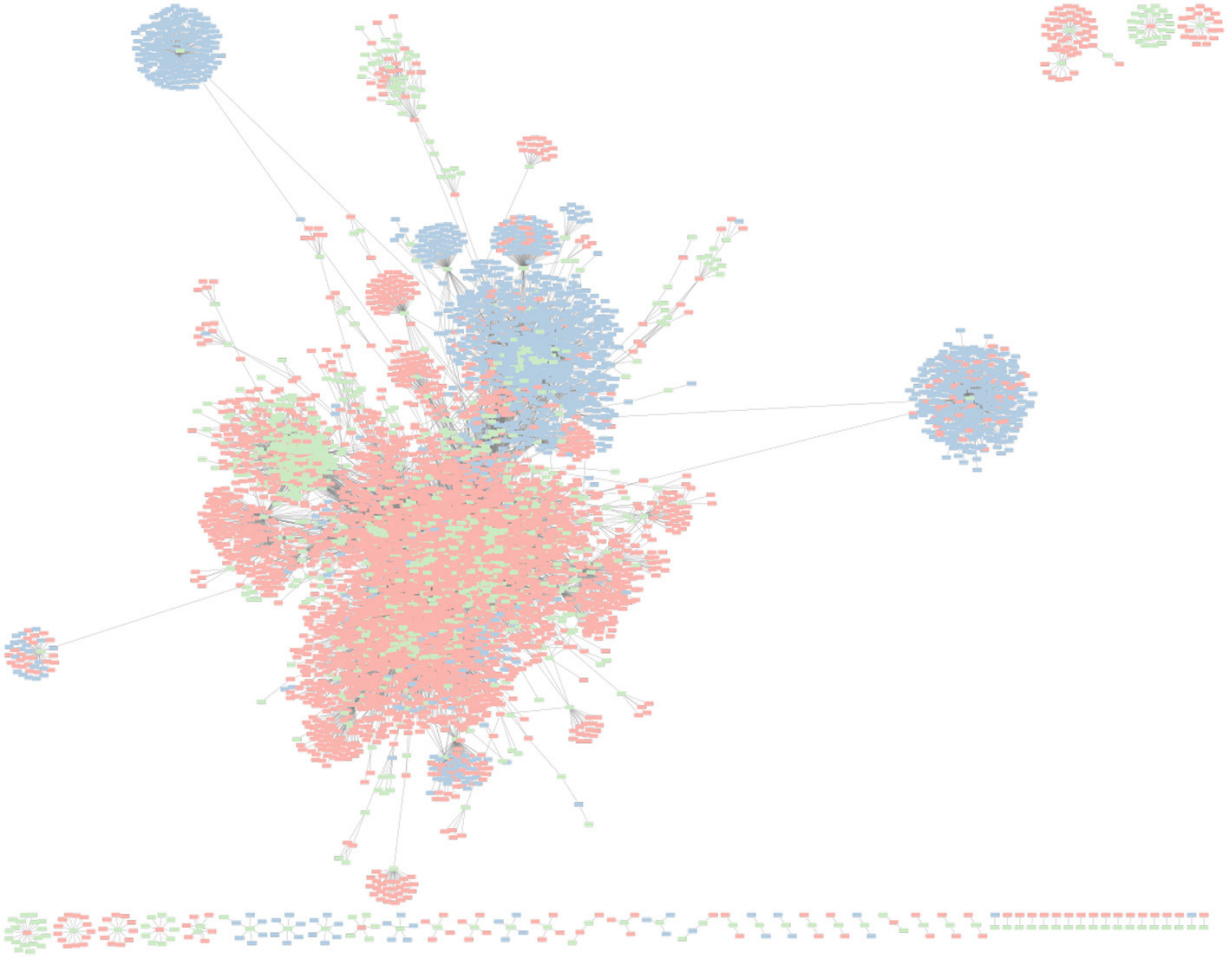
Network graph representing *K. pneumoniae* associated CRISPR–Cas spacers and their corresponding putative MGE (e.g., phage, plasmids) targets. Green nodes represent unique CRISPR–Cas spacer sequences found in *K. pneumonia* reference genomes. Red nodes represent phage genomes. Blue nodes represent plasmid genomes. An edge between spacer and MGE nodes is depicted when a spacer has a corresponding protospacer in a given MGE.

To further explore the association between *K. pneumoniae* and their invading MGEs, we constructed a spacer-MGE association network (Figure 7). Each identified CRISPR spacer is represented as a spacer node, and an edge is constructed if a matching MGE contains a complementary protospacer. Spacer nodes was found to have a mean degree of 11.88. In some cases, a single spacer node was found to have a degree of 432; meaning that a single spacer was able to target 432 putative MGE invaders. High degree of a spacer node suggests that a given spacer can target conserved regions across various MGE genome targets.

Interestingly, MGE targets (phages in particular) of *K. pneumoniae* CRISPR–Cas systems were found to be targeted by multiple spacers. Phages were found to have an average degree of 6.86, meaning on average each phage target had about 6–7 protospacers complementary to a matching CRISPR spacer. The maximum degree of any phage was 155. In contrast, plasmids had an average degree of 4.30, and max degree of 58. CRISPR–Cas systems have been shown to independently develop immunity to the sample MGE at different protospacer locations.

## 4. Discussions

Here we presented an evaluation of CRISPR–Cas system in ESKAPE+C pathogens to better understand their distribution of CRISPR–Cas systems, their interactions with MGEs, and their correlation with AMR genes. Given the nature of ESKAPE+C pathogens and emergence of MDR bacteria, the focus on mechanisms of antimicrobial resistance and factors that contribute to bacterial virulence has become increasingly important in order to advance methods and strategies to mitigate MDR bacterial infections. Our exploration of ESKAPE+C pathogens and their potential MGE invaders sheds light on mechanisms of evolution that drive horizontal gene transfer of AMR genes, as well as provides a resource of known ESKAPE+C pathogen host-MGE associations that can help inform scientist and policy makers in devising strategies to combat ESKAPE+C pathogens (e.g., phage therapy). While studies have shown that CRISPR–Cas systems affect Host-MGE interactions, and in turn horizontal gene transfer (Brito, 2021), here we perform the first in-depth analysis of the ESKAPE+C species in context of their CRISPR–Cas systems and AMR genes to our knowledge. Our work provides a unique perspective of the ESKAPE+C pathogens, AMR genes at the pangenomic level, CRISPR–Cas systems, and their interactions with MGEs. In addition, we have made available (with visualization) all identified CRISPR–Cas systems in the pangenomes of the CRISPR+C pathogens, through a website.

Our evaluation CRISPR–Cas systems in ESKAPE+C pathogens not only revealed that the prevalence of CRISPR–Cas systems is correlated with the median genome size (in most cases), but also showed a statistically significant relationship between the presence of CRISPR–Cas systems and the lack of AMR genes in *A. baumannii, E. faecium, K. pneumoniae*, and *S. aureus*. Of the ESKAPE+C species, only *K. pneumoniae, Enterococcus* spp., and *C. difficile* was not found to have a correlation between CRISPR–Cas presence and lack of AMR genes. While we excluded *Enterococcus* spp. from our analysis, *K. pneumoniae* lack of correlation was most likely attributed to the presence of its CRISPR–Cas system located on an associated plasmid which also contained AMR genes, and *C. difficile* did not have a correlation due to every genome evaluated containing a CRISPR–Cas system. By evaluating the relationship between CRISPR–Cas systems and their effects on evolutionary processes, such as horizontal gene transfer of AMR genes, we were able to show contributing underlying factors that affect ESKAPE+C pathogen virulence.

Focusing our analysis on interactions between host genomes of ESKAPE+C species and their putative CRISPR targets, we were able to resolve host-MGE interaction dynamics. We showed that in *K. pneumoniae*, CRISPR spacers were enriched for targeting of phages in comparison to plasmids. This difference was also reflected in the number of protospacers found in protospacer containing genomes, where phages on average had more protospacers than their plasmid counterparts. Interestingly, we found that there were many instances of spacers targeting conserved regions across many MGEs, and also many MGEs being targeted at various loci by CRISPR spacers. These instances of MGE with multiple protospacers, coupled with many CRISPRs independently gaining unique spacers to target the same MGE, may suggest that CRISPR–Cas systems develop biases toward targeting certain invading MGEs, or that certain MGEs remain a constant and continuous threat to certain genomes, and thus it is more likely for an active CRISPR–Cas system to gain spacers targeting these MGEs.

While we acknowledge that our analysis is limited by the reference databases used (e.g., genome reference database, AMR database, MGE databases), and that these databases may be incomplete, the findings of this paper helps provide invaluable insights into ESKAPE+C evolutionary processes, as well as helps establish a resource of known MGE targets by ESKAPE+C species. A significant limiting factor to the understanding microbiome dynamics and host-MGE interactions is the lack of an aggregated and curated MGE database. In order to better understand microbiome dynamics which influence health and disease, it is imperative that researchers begin cataloging MGE assemblies along with their prokaryotic counterparts. By providing the availability of these known host-MGE interactions, we help inform future research of potential bacterial phages that should be used with caution in potential phage cocktails in phage therapy applications for ESKAPE+C species. Additionally, statistical significance between the presence of CRISPR–Cas systems and the absence of AMR genes only suggests correlation. Further research is necessary to validate causality, but such experiments remain difficult to design in metagenome studies in a reproducible and controlled fashion.

Nevertheless, research into ESKAPE+C pathogens remains an ever-important area of research due to the increasing need for alternative methods to deal with MDR bacteria. Basic research into understanding how ESKAPE+C pathogens evolve, adapt, and become virulent are important steps into developing alternative treatment options such as phage therapy.

## Data Availability Statement

The original contributions presented in the study are included in the article/supplementary material, further inquiries can be directed to the corresponding author/s.

## Author Contributions

YY conceived and planned the experiments. KM, TL, and YY carried out the experiments. All contributed to the interpretation of the results and participated in writing the manuscript.

## Funding

This work was supported by NIH grant 1R01AI143254 and NSF 2025451.

## Conflict of Interest

The authors declare that the research was conducted in the absence of any commercial or financial relationships that could be construed as a potential conflict of interest.

## Publisher's Note

All claims expressed in this article are solely those of the authors and do not necessarily represent those of their affiliated organizations, or those of the publisher, the editors and the reviewers. Any product that may be evaluated in this article, or claim that may be made by its manufacturer, is not guaranteed or endorsed by the publisher.
